# Genetic variation in adaptive traits and seed transfer zones for *Pseudoroegneria spicata* (bluebunch wheatgrass) in the northwestern United States

**DOI:** 10.1111/eva.12077

**Published:** 2013-06-06

**Authors:** John Bradley St. Clair, Francis F Kilkenny, Richard C Johnson, Nancy L Shaw, George Weaver

**Affiliations:** 1 United States Department of Agriculture Forest Service, Pacific Northwest Research StationCorvallis, OR, USA; 2 United States Department of Agriculture Agricultural Research Service, Plant Germplasm Research and TestingPullman, WA, USA; 3 United States Department of Agriculture Forest Service, Rocky Mountain Research StationBoise, ID, USA; 4 Department of Statistics, Oregon State UniversityCorvallis, OR, USA

**Keywords:** climate change, genecology, plant adaptation, *Pseudoroegneria spicata*, seed transfer, seed zones

## Abstract

A genecological approach was used to explore genetic variation in adaptive traits in *Pseudoroegneria spicata*, a key restoration grass, in the intermountain western United States. Common garden experiments were established at three contrasting sites with seedlings from two maternal parents from each of 114 populations along with five commercial releases commonly used in restoration. Traits associated with size, flowering phenology, and leaf width varied considerably among populations and were moderately correlated with the climates of the seed sources. *Pseudoroegneria spicata* populations from warm, arid source environments were smaller with earlier phenology and had relatively narrow leaves than those from mild climates with cool summers, warm winters, low seasonal temperature differentials, high precipitation, and low aridity. Later phenology was generally associated with populations from colder climates. Releases were larger and more fecund than most of the native ecotypes, but were similar to native populations near their source of origin. Differences among native populations associated with source climates that are logical for survival, growth, and reproduction indicate that genetic variation across the landscape is adaptive and should be considered during restoration. Results were used to delineate seed transfer zones and population movement guidelines to ensure adapted plant materials for restoration activities.

## Introduction

Genecology is the study of intraspecific genetic variation in relation to source environments, a term first used by Swedish evolutionary biologist Göte Turesson (1923). Strong and interpretable correlations between geographic genetic variation as revealed in common garden studies and the environments in which populations evolved suggest adaptation as determined by natural selection (Heslop-Harrison 1964; Endler 1986). Presumably, parents with heritable characteristics that allowed them to grow, survive, and reproduce in a past environment at a source location produced progeny that express those characteristics when grown together in a common environment. Natural selection is suggested when populations differ in putative adaptive traits, those differences are correlated with characteristics of the source environments, and the relationship between a trait and an environmental character is logical for growth, survival, or reproduction.

One practical advantage of genecology studies is the ability to evaluate a large number of populations from a wide range of source environments in one or a few easily accessible common gardens. By relating traits to climates or other environmental variables, researchers are able to map adaptive traits across the landscape (Campbell 1986; Sorensen 1992; Rehfeldt 1994a; Erickson et al. 2004; St. Clair et al. 2005). Large numbers of populations are particularly useful in highly heterogeneous environments such as in the mountainous terrain of western North America. These maps may be used to delineate seed transfer zones or population movement guidelines to ensure that germplasm used in restoration or revegetation is adapted to the site; however, population movement guidelines based on genecology studies assume that local populations are best adapted to local environments and less well adapted as you move to different environments. Nevertheless, a large number of studies have shown that in most cases, locally derived germplasm is at least approximately best adapted to local climatic and environmental conditions (Hufford and Mazer 2003; Savolainen et al. 2007; Johnson et al. 2010a). Genecology studies also assume that the most important adaptive traits have been evaluated. This assumption may be addressed by measuring many putative adaptive traits, by measuring traits that are strongly correlated with several physiological or morphological traits important for adaptation such as phenology, or by measuring traits that integrate physiology or stress response over time such as growth and survival. Traits that may not have been evaluated – disease and insect resistance, for example – should be considered when delineating seed zones or population movement guidelines. Although a large number of traits may complicate interpretations from genecology studies, multivariate statistics such as principle component analysis may be used to reduce the number of interacting factors and make it easier to consider several uncorrelated factors when delineating seed zones (Campbell 1986; St. Clair et al. 2005).

The assumption of local adaptation is best tested using reciprocal transplant studies in which samples of genotypes from local populations are compared with samples from distant populations in a series of common garden test sites, preferably in native habitats (Kawecki and Ebert 2004). Most reciprocal transplant studies, however, have their own limitations. First, the challenge of testing a large number of populations over a large number of locations limits most reciprocal transplant studies to only a few populations evaluated in a few test environments, making it difficult to interpolate between locations to adequately characterize population response functions or transfer functions, as well as produce maps of adaptive variation, a particular problem in areas of highly heterogeneous environments (but see Wang et al. 2010 for an exception using a large *Pinus contorta* provenance test). Second, although reciprocal transplant studies can provide a test of adaptation, to do so implies following growth, survival, and reproduction over a generation or more, which can be difficult for long-lived plant species. Third, reciprocal transplant studies often do not consider which specific traits are important for fitness and adaptation and, thus, useful for selection of populations or individuals.

Genecological studies have been used for some time to delineate seed zones and population movement guidelines for commercial forest trees (e.g., Campbell 1986; Sorensen 1992; Beaulieu et al. 2004; St. Clair et al. 2005). More recently, seed zones have been delineated using a genecological approach for several nontree native plants used in restoration, including *Elymus glaucus* (Erickson et al. 2004; Kitzmiller 2009), *Bromus carinatus* (Kitzmiller 2009; Johnson et al. 2010a,b), *Bromus orcuttianus* (Kitzmiller 2009), *Festuca roemeri* (Wilson et al. 2008), and *Holodiscus discolor* (Horning et al. 2010). In this study, we use a genecological approach to map geographic genetic variation in adaptive traits and delineate seed zones for *P. spicata* (Pursh) A. Löve (Poaceae, bluebunch wheatgrass), a species used widely in restoration, but for which little is known about adaptive geographic variation. *P. spicata* is a cool-season, outcrossing perennial bunchgrass of semi-arid regions of western North America (Cronquist et al. 1977; Zlatnik 1999; Carlson 2007; Monsen et al. 2004). It is found in a wide variety of habitats in inland regions from Alaska and Saskatchewan to California and west Texas. It is a dominant species of many grasslands of the intermountain western United States, occurring at elevations from 150 to 3,000 m. It is best adapted where precipitation is 250–500 mm, but occurs in areas with as little as 200 mm. *Pseudoroegneria spicata* is fairly common on sandy and clayey soils, but also grows well on thin, rocky, and gravelly soils. It is not tolerant of alkaline or saline soils or excessive soil moisture. Two subspecies or forms are recognized: *P. spicata* ssp. *spicata* and *P. spicata* ssp. *inerme* (beardless bluebunch wheatgrass) (Cronquist et al. 1977; Zlatnik 1999; Carlson 2007). The only difference between them is the presence or absence of divergent awns. The two subspecies are conterminous and hybridize (Daubenmire 1939). *Pseudoroegneria spicata* is predominately diploid (2n = 14), although autotetraploid forms (4n = 28) may be found in eastern Washington and northern Idaho (Hartung 1946).

*Pseudoroegneria spicata* is an important forage species for both livestock and wildlife (Zlatnik 1999). It has been reduced over extensive areas by overgrazing, as well as by mechanical disturbance and frequent fires (Monsen et al. 2004). Although bluebunch wheatgrass recovers well after fires, invasion by annual weeds, particularly *Bromus tectorum* (cheatgrass), has hindered re-establishment (Zlatnik 1999; Monsen et al. 2004). Although not as resistant to grazing as some species, once established, *P. spicata* stands have extensive drought-resistant root systems that can compete with and suppress the spread of exotic weeds, including *B. tectorum* (Zlatnik 1999). *Pseudoroegneria spicata* is an important species for restoration, although many restoration projects rely upon only a few commercially available seed lots that have been selected, reproduced, marketed, and made available for restoration and revegetation (referred to as releases). Although the commercial releases have proven useful over a wide range of environments, Monsen et al. (2004) indicate that few have proven to be well adapted across the Great Basin and that selection and use of specifically adapted ecotypes is recommended.

The wide distribution across a diverse range of habitats and large morphological variation suggests that *P. spicata* is genetically variable, and that much of that variation may be adaptive. The objectives of this study are (i) to explore genetic variation in putative adaptive traits in *P. spicata* from a wide range of source environments in the inland Pacific Northwest and northern Great Basin; (ii) to explore relationships between genetic variation in putative adaptive traits and the climates of seed sources; (iii) to delineate seed zones and population movement guidelines; and (iv) to compare commercially available releases of *P. spicata* to native sources.

## Materials and methods

### Population sampling

**Figure 1:**
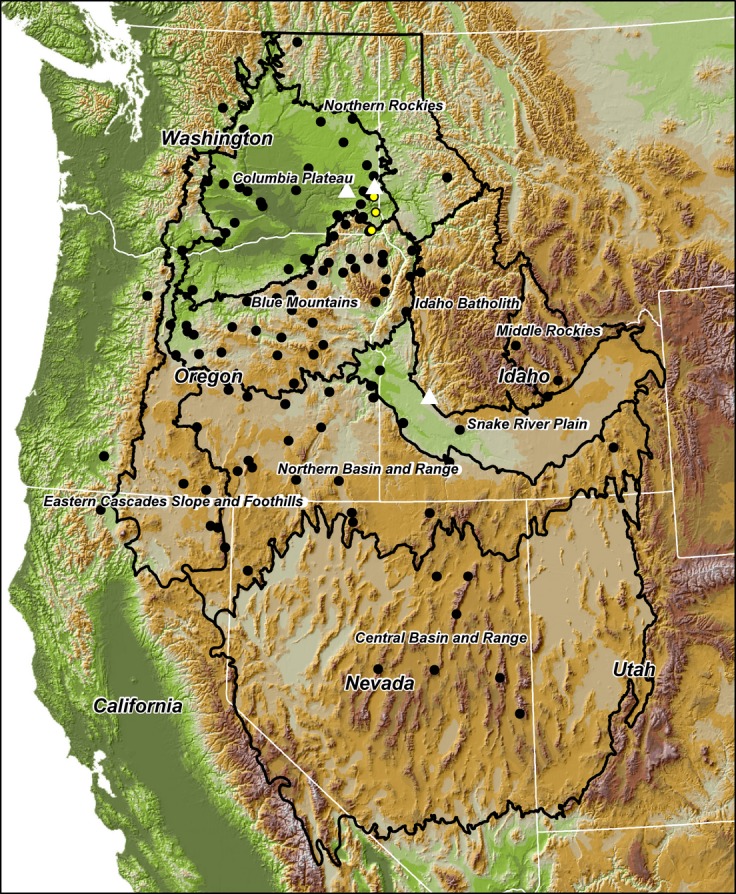
Study area with level III ecoregions (Omernik 1987) labeled and locations of 114 *Pseudoroegneria spicata* native collections (•), original locations of three commercial releases (○), and three test sites (Δ).

Wildland seed was collected in the summer of 2005 from *P. spicata* plants in 114 populations located throughout the inland Pacific Northwest and northern Great Basin (Fig. 1). The collection locations reflected the range of environments and climatic conditions over which *P. spicata* occurs, ranging from 230 to 2460 m in elevation, 210 to 1870 mm in annual precipitation, and 3.2 to 11.5°C in mean annual temperature. The populations are predominately from six level III ecoregions (Omernik 1987): central Basin and Range, northern Basin and Range, Blue Mountains, Columbia Plateau, Eastern Cascades Slopes and Foothills, and Snake River Plain. At each location, seed was collected and stored by individual maternal parent plants, separated by a minimum of 5 m to minimize relatedness within locations. The progeny from two parent plants at each location, each of which can be referred to as a family, were used in this study to represent each of the 114 populations. This approach provided a pooled estimate of variance among families within populations, which was used as the error term to test differences among populations for plant traits. The sampling scheme of many populations and few families per population sacrifices precise estimates of population means for a better estimate of the response function across the landscape, an approach that is particularly valuable across highly heterogeneous environments such as in the mountainous western United States. Latitude, longitude, and elevation were recorded for each seed source location using geographic positioning instrumentation. Climate normals for the period 1971–2000 for each seed source location were obtained using ClimateWNA (Wang et al. 2012; http://www.genetics.forestry.ubc.ca/cfcg/ClimateWNA/ClimateWNA.html). Climate variables included monthly, seasonal, and annual variables for precipitation and mean, minimum, and maximum temperature, as well as other derived variables related to chilling and growing degree days, aridity, frost, precipitation as snow, and extreme temperatures over 30 years.

The releases Whitmar, Goldar, Anatone, P-7, and Secar were included in the study for comparisons with the wildland populations. The releases were obtained from germplasm collections at the United States Department of Agriculture Natural Resource Conservation Service Plant Materials Center and the Western Regional Plant Introduction Station, both in Pullman, Washington.

### Experimental design

The common garden experiments were established in September 2006 at three contrasting test sites: the Central Ferry Research Farm (46°40′9″N, 117°45′21″W), the Pullman Plant Materials Center (46°43′9″N, 117°8′29″W), and the Lucky Peak Nursery (43°34′57″N, 115°29′36″W) (Fig. 1). Central Ferry (CF) is located in the Snake River Canyon in southeastern Washington State at about 200 m a.s.l. The climate at CF is relatively hot and dry, with a mean annual temperature of 11.4°C, mean temperature in the coldest month of 0.7°C, and mean annual precipitation of 340 mm. Pullman (PU) is also located in southeastern Washington State, about 50 km east of CF at a higher elevation of 750 m a.s.l., with a correspondingly cooler and wetter climate. Mean annual temperature at PU is 8.6°C, mean annual cold month temperature is −1.1°C, and mean annual precipitation is 610 mm. Lucky Peak (LP) is located about 20 km east of Boise, Idaho, at an elevation of 975 m a.s.l. The climate is dry with a mean annual precipitation of 390 mm. The mean annual temperature is 10.1°C, intermediate between CF and PU, although it is colder in the winter with a mean coldest month temperature of −1.8°C.

Seeds from the 228 families (two families from each of 114 populations) and the five commercial releases were sown in July 2006 in germination boxes (13.3 cm long × 12.7 cm wide × 3.5 cm deep) containing water-saturated vermiculate. The boxes were placed at room temperature (∼20°C) in a laboratory in PU and seeds allowed to germinate. Germinants were planted into ‘books’ of five 2.5 cm^2^ × 11.4 cm long plastic cells filled with potting soil. The plants were placed outside during the summer for approximately 8 weeks and watered and fertilized as needed to promote optimal growth. The test sites were cultivated prior to transplanting to provide a suitable planting bed. Seedlings were transplanted by hand on September 12 at LP, September 26 at PU, and September 27 at CF. Plant spacing was 0.6 m × 1.8 m. At CF and LP, supplemental irrigation (∼6 mm) was applied prior to planting to ensure initial survival. Soil moisture was excellent at PU, and supplemental irrigation was not required. The experimental design at each test site was a randomized block design. A single plant from each of the 228 families and two plants from each of the five releases were randomly assigned to planting spots within each of six replicate blocks at a site. Thus, each of the 114 populations in the study was represented by six individuals from each of two families, and each of the five releases was represented by twelve individuals. Each test site was surrounded by a single border row. The total number of study plants at each test site was 1428.

### Measurements and statistical analyses

All plants were measured for a variety of growth, fecundity, morphology, and phenology traits during the spring and summer of 2007 and 2008 (Table 1). In addition, germination tests were carried out in winter 2009 on seed collected in late summer 2008 from two randomly selected plants of each family at each test site. For the germination tests, 25 seeds from each plant sampled in the common gardens were placed in a small petri dish (35 mm diameter × 18 mm deep) with moist filter paper containing a solution of 0.7% Captan 50 fungicide and 0.2% potassium nitrate. The petri dishes were placed in a controlled environment chamber with a daytime temperature of 25°C for 8 h and a nighttime temperature of 15°C for 16 h and no lights. The number of germinants was recorded every 2 days over the next 4 weeks. The procedure was later replicated with another 25 seeds from each plant. Rates of germination were determined as days to 50% germination using a probit model fitted separately for each plant using the procedures of Campbell and Sorensen (1979).

**Table 1:** Descriptions of traits measured on *Pseudoroegneria spicata* plants grown in common environments at three sites in the inland Pacific Northwest

Trait	Abbreviation	Description
Dry weight	DRYWT	Plant dry weight (g) cut at 5 cm aboveground at time of seed maturity after oven drying
Crown width	CW	Average width (cm) of base of plant measured after harvest in widest direction and perpendicular to widest direction
Plant height	HT	Height (cm) of plant including spikes at time of first anthesis
Regrowth dry weight	REGRWT	Plant dry weight (g) of regrowth after harvest cut at 5 cm aboveground in fall
Regrowth height	REGRHT	Height (cm) of plant regrowth after harvest measured in fall
Inflorescence number	INFLNO	Number of inflorescences at time of first anthesis
Spikelet number	SPKNO	Number of spikelets on a representative* inflorescence
Culm length	CULMLNG	Length (cm) of culm on representative* inflorescence from base of plant to beginning of spike
Spike length	SPKLNG	Length (cm) of spike on representative* inflorescence
Inflorescence length	INFLLNG	Length (cm) of representative* inflorescence measured from base of plant (equals culm plus spike lengths)
Leaf length	LFLNG	Length (cm) of representative* upper, fully emerged leaf
Leaf width	LFWD	Width (cm) of representative* upper, fully emerged leaf measured on the same leaf as leaf length
Leaf length/width ratio	LFRATIO	Ratio of leaf length to width
Leaf color	LFCOL	Color of an upper, fully emerged leaf using a scale of 1 = yellow green to 9 = dark blue green
Leaf pubescence	LFPUB	Pubescence on an upper, fully emerged leaf using a scale of 1 = no pubescence to 9 = high density of pubescence
Plant habit	HABIT	Characteristic form or mode of growth of plant using a scale of 1 = prostate to 9 = upright
Awn length	AWNS	Length of awns using a scale of 0 = no awns to 9 = very long awns
Date of first heading	HEAD	Day of year when first inflorescence emerges from sheath
Date of first anthesis	BLOOM	Day of year when anthers first arise from spikelets
Date of seed maturation	MATURE	Day of year when seeds are mature as indicated by start of shatter or hardening of seeds on the spikelet
Rate of germination	GERM	Days to 50% germination for seed collected at test sites in 2008
Seed weight	SEEDWT	Weight of 100 seeds in g for seed collected at test sites in 2008

* Representative leaves and inflorescences are randomly sampled from a plant excluding the very tallest and shortest.

The general statistical methodology followed the procedures of Campbell (1986) for describing and mapping genetic variation across the landscape. Analysis of variance was performed to test for significant differences in measured traits among the main effects of years, test sites and populations, and their interactions, using PROC MIXED of the SAS statistical package (SAS Institute 2013). Replicates were nested within site, and families were nested within populations. Interactions between pairs of test sites in each year and between years at each test site were further explored by considering the magnitude of the correlations of population means. Strong correlations indicate that performance was similar between test sites or between years.

For purposes of exploring variation in putative adaptive traits, traits measured at different test sites and in different years were considered to be different traits. Variance components for populations, families within populations, and error were determined for each trait at each site and in each year using PROC MIXED. Traits that had high percentages of total phenotypic variance attributed to populations were considered promising traits for further exploration of potentially adaptive relationships with climates of source locations.

Many traits were correlated and might be considered as the same or similar traits. Principal component analysis (PCA) was carried out to reduce the number of traits to fewer uncorrelated multivariate traits. The potential number of traits for PCA was 123 (20 traits measured at three test sites in 2 years plus germination and weight from seed collected at three test sites in 1 year). To reduce the number of traits prior to PCA, traits with low population variance (defined as <10% of total phenotypic variance) were dropped from the analysis, and traits that were at least moderately correlated (defined as *r* ≥ 0.40) between sites or years were averaged across sites or years or both. PCA was carried out using PROC PRINCOMP in SAS using the correlation matrix of population means of the reduced set of traits.

The relationship between traits and source environments was evaluated by considering correlations between traits and the climates of seed sources and by multiple regressions of trait values on climatic variables. Regressions models were chosen using the *R*^2^ selection procedure in PROC REG in SAS, which ranks parameter sets by maximal coefficients of determination (R^2^). Additionally, AIC values were calculated and used to discard overparameterized models by selecting models with the lowest AIC value. Regressions were carried out both with individual traits and with multivariate traits as determined by principal components (PCs). *R*^2^ values for the regression provide an estimate of the strength of the relationship between a given individual or multivariate trait and the multivariate climate. Although not indicative of local adaptation, traits with high population variation and strong relationships with climate may be considered to be more important for adaptation, particularly if the relationship between a trait and climate makes sense for survival, growth, or reproduction.

The regression models were used to map traits across the landscape using the grid algebra function of the ArcGIS 9.3 Spatial Analyst extension (ESRI 2008). Each grid location (0.057 × 0.057 decimal degrees or ∼6 × 6 km) in the geographic information system (GIS) was assigned values for climatic variables using ClimateWNA, which in turn were converted to trait values by multiplying each climatic variable by the respective regression coefficient and summing the results. To avoid extrapolating beyond the study area, map boundaries were limited to the area sampled including the six level III ecoregions and areas in adjacent ecoregions in Idaho and northeastern Washington. Some areas within those boundaries, however, might be beyond the climatic or realized niche of the species. In particular, while collecting seed, we did not find *P. spicata* populations in the low-elevation arid regions of western Nevada and northeastern Utah. Seed zones delineating similar genetic types (i.e., ecotypes) were created by classifying the first three PCs into two or three categories and then overlapping the resulting maps (similar to the procedures of St. Clair et al. 2005). PC 1 scores were mapped across the landscape into high, medium, and low categories with the boundaries between categories being plus or minus one standard deviation from the overall mean of sampled populations. PC 2 and PC 3 scores were mapped across the landscape into high and low categories with the boundaries at the mean of zero.

## Results

### Variation in plant traits

Most traits were significantly different among test sites (Table 2). Plants at the warmer CF site were much larger as measured by biomass and crown width than plants at the other two sites, while plants at the cooler PU site were smallest (Table 3). Plants at CF were also considerably more fecund than plants at the other two sites, with more than three times the number of inflorescences than those at PU. Leaves were relatively long and narrow at CF. Plants at CF had the earliest phenology as evidenced by earlier dates of heading, anthesis, and seed maturation.

**Table 2:** Results of analyses of variance for traits of 114 *Pseudoroegneria spicata* populations measured in 2 years at three common garden test sites

	*F*-value					
	
Trait†	Population	Test site	Year	Population × test site	Population × year	Test site × year
DRYWT	15.51**	80.70**	2690.48**	5.59**	9.51**	939.13**
CW	15.05**	89.62**	8923.33**	2.75**	5.34**	1003.37**
HT	6.95**	94.09**	1.98	2.37**	1.94**	71.47**
REGRWT	11.58**	39.72**	2876.20**	2.35**	9.72**	969.53**
REGRHT	6.80**	72.18**	0.00	3.16**	1.36**	105.64**
INFLNO	8.73**	119.48**	4044.67**	3.45**	6.60**	2087.96**
CULMLNG	6.18**	1031.27**	191.42**	5.09**	2.07**	135.35**
SPKLNG	0.82	27.05**	10.92**	0.91	1.06	1.49
INFLLNG	1.89**	391.18**	0.35	1.19*	1.17	4.60*
SPKNO	8.78**	266.57**	372.33**	1.54**	1.96**	70.81**
LFLNG	6.05**	73.25**	338.85**	1.86**	2.11**	481.89**
LFWD	3.34**	0.56	259.51**	0.68	0.67	126.41**
LFRATIO	12.22**	14.16**	1.27	1.77**	1.55**	71.12**
LFCOL	4.97**	1.23	64.80**	1.21*	1.36**	13.11**
LFPUB	10.29**	0.03	1710.47**	3.16**	1.78**	293.52**
HABIT	5.27**	12.63**	1.27	2.39**	2.53**	143.55**
AWNS	18.05**	0.21	17.69**	1.43**	1.21	1.05
HEAD	6.28**	784.14**	1805.41**	1.75**	2.05**	424.14**
BLOOM	7.23**	1615.44**	3232.00**	1.30**	1.85**	780.82**
MATURE	2.66**	376.15**	138.68**	1.39**	1.98**	217.15**
GERM‡	2.82**	34.60**	–	1.41**	–	–
SEEDWT‡	4.32**	12.35**	–	3.13**	–	–

Statistical significance indicated as

* *P* = 0.05−0.01 or

** *P* < 0.01.

† See Table 1 for trait codes and descriptions.

‡ Germination and seed weight traits were measured in a single year only.

**Table 3:** Overall trait means for 114 *Pseudoroegneria spicata* populations measured at three common garden test sites and in two different years

Trait*	Test site			Year	
	
	Central Ferry	Lucky Peak	Pullman	2007	2008
DRYWT	135	54	36	42	108
CW	10.4	7.2	7.0	5.5	10.9
HT	30	54	25	36	37
REGRWT	25	37	11	9	40
REGRHT	20	9	14	14	14
INFLNO	159	59	44	28	146
SPKNO	11.9	9.6	10.0	9.9	11.1
CULMLNG	59	21	49	41	44
SPKLNG	21	14	14	18	15
INFLLNG	80	35	63	59	60
LFLNG	17.0	12.5	12.6	14.7	13.3
LFWD	0.41	0.39	0.58	0.55	0.38
LFRATIO	45	34	35	38	38
LFCOL	3.2	3.2	2.8	3.3	2.9
LFPUB	4.1	4.2	4.2	4.8	3.5
HABIT	6.3	5.7	6.8	6.3	6.3
AWNS	4.6	4.7	4.6	4.5	4.7
HEAD	129	139	145	133	142
BLOOM	141	152	165	148	158
MATURE	179	192	202	190	193
GERM†	6.09	6.82	6.19	–	6.39
SEEDWT†	0.41	0.40	0.38	–	0.40

* See Table 1 for trait codes and descriptions.

† Germination and seed weight traits were measured in a single year only.

Most traits differed significantly as measured in different years (Table 2). As expected, plants in their second full growing season (in 2008) were considerably larger and more fecund (Table 3) than plants after their first growing season. Phenology was earlier in 2007 than in 2008, likely due to a cooler spring in 2008 (2.2°C cooler averaged over the three sites).

As is common with open-pollinated species, most of the phenotypic variation in plants grown in a common environment was attributed to variation in individual plants within families. Population variation, however, was large and statistically significant (Table 2), in some cases more than 40% of the total phenotypic variation (Table 4). Variation among families within populations was small, averaging only about 3.5% of total phenotypic variation. Traits with high levels of population variance included dry weight, crown width, inflorescence number, leaf height/width ratio, awn length, and dates of anthesis. Traits with low population variation included date of maturation, plant height, leaf length, leaf color, inflorescence length, and spike length.

**Table 4:** Percentages of total phenotypic variance attributed to populations for traits in each of three test sites in two different years and averaged over all test sites and years

Trait*	Central Ferry		Lucky Peak		Pullman		Overall
		
	2007	2008	2007	2008	2007	2008	
DRYWT	45	47	21	36	31	27	35
CW	32	43	23	33	28	30	32
HT	18	8	12	29	13	12	15
REGRWT	18	31	23	36	21	25	26
REGRHT	17	26	22	34	19	19	23
INFLNO	44	35	33	19	32	24	31
SPKNO	17	24	23	23	36	25	25
CULMLNG	34	21	18	9	28	28	23
SPKLNG	0	16	13	20	27	12	15
INFLLNG	1	19	17	14	29	25	18
LFLNG	16	26	19	14	16	19	18
LFWD	21	13	56	33	0	9	22
LFRATIO	25	38	36	37	15	37	32
LFCOL	34	8	37	30	3	5	19
LFPUB	32	23	26	12	39	26	26
HABIT	13	25	28	28	7	22	21
AWNS	41	27	55	55	45	42	44
HEAD	39	14	31	35	20	12	25
BLOOM	22	1	44	47	28	41	30
MATURE	18	0	13	29	9	4	12
GERM†	–	26	–	26	–	13	22
SEEDWT†	–	27	–	35	–	29	30

* See Table 1 for trait codes and descriptions.

† Germination and seed weight traits were measured in 2008 only.

The interactions of populations with test sites and populations with year were statistically significant for nearly all traits, indicating that population responses differed among test sites and between years; however, *F*-values were generally not large compared with main effects (Table 2). Correlations of population means between pairs of test sites and between years were positive and generally strong to moderate (Table S1), indicating that population responses were similar at different test sites and in different years despite statistical significance of the population × test site and population × year interactions.

### Relationships between traits and principal component analysis

As expected, correlations were found among numerous plant traits that may reflect similar underlying characteristics or genetic pathways. For example, the correlation of population means averaged over all sites and years for dry weight with crown width was 0.93, and that for dry weight with inflorescence number was 0.90. Interestingly, dry weight and plant height were not as strongly correlated (*r* = 0.56). Correlations among size and fecundity measures indicated that populations with high dry weight at all sites and in both years had high seed production as measured by inflorescence number. Phenology traits were also positively correlated, although dates of heading were more highly correlated with dates of anthesis (*r* = 0.81) than with dates of seed maturation (*r* = 0.31). Other traits were not strongly correlated and appeared more independent. For example, dates of heading were weakly correlated with dry weights (*r* = −0.06) and with leaf ratio (*r* = −0.22). The large number of traits and the presence of both weak and strong correlations suggest that PCA may be useful to reduce the number of traits to a few uncorrelated multivariate traits that may be easily interpreted.

Principal component analysis was carried out on 35 traits after combining traits that were correlated (defined as *r* > 0.40) and eliminating traits with low population variation (<10% of the total phenotypic variance). The PCA extracted three PC that accounted for more than half of the population variance in the original traits (Table 5). Each subsequent principal component explained 6% or less of the variance. Given the relatively small variation explained by PCs beyond the first three, only the first three components were considered for regression modeling, mapping, and seed zone delineation. The first three PCs were interpreted by considering the eigenvector loading coefficients and the correlations with traits averaged over all sites and years (Table S2). PC 1 represented increased vigor as indicated by strong correlations with dry weight, crown width, inflorescence number, and other size traits. Populations with high values for PC 1 were larger, more fecund, and with greater regrowth after harvest in the summer. They also tended to be more prostrate and greener. PC 2 was strongly represented by phenology with high values indicating later phenology, but also generally taller plants. PC 3 was most representative of leaf morphology and plant habit. Populations with higher values of PC 3 had relatively narrow leaves with a more upright plant habit.

**Table 5:** Results of principle component analysis of 35 traits measured on 114 *Pseudoroegneria spicata* populations grown three common garden test sites

Principle component	Eigenvalue	Percent variation explained	Cumulative percent variation	Interpretation*
1	9.96	28	28	Larger size, more inflorescences
2	5.57	16	44	Later heading and anthesis
3	3.11	9	53	Narrower leaves
4	2.19	6	60	Earlier heading but later seed maturation, slower germination
5	1.93	6	65	Longer awns, more upright
6	1.56	4	70	Greener and more pubescent leaves

* Interpretation based on examination of eigenvector loadings and correlations of PCs with individual traits.

### Relationships of traits with source environments and geographic patterns of variation

Genecological relationships of traits with source environments were evaluated by considering the correlations and regressions for the first three PCs and for several individual traits. For individual traits, only results for dry weight, date of heading, and leaf form are presented; these traits had high population variation (Table 4) and were more strongly correlated with climatic variables than many other variables (Table 6) and, thus, are likely to be important for adaptation. These traits were also correlated with the first three PCs (Table S2). Correlations and regressions of individual traits with climate were based on populations means averaged over all three sites and both years.

**Table 6:** Correlations* of principle components and selected individual traits† with climate and geography of source locations

Climate/geography‡	PC1	PC2	PC3	DRYWT	HT	HEAD	LFRATIO	LFCOL	LFPUB	HABIT	AWNS	GERM
MAT	0.08	−0.20	0.48	0.09	0.09	−0.40	0.53	−0.16	0.07	0.05	−0.18	0.03
MWMT	−0.14	−0.18	0.47	−0.14	0.00	−0.40	0.55	−0.31	0.15	0.14	−0.32	0.17
MCMT	0.31	−0.05	0.31	0.29	0.25	−0.14	0.29	0.10	−0.05	−0.03	0.17	−0.12
TD	−0.42	−0.14	0.20	−0.40	−0.22	−0.27	0.30	−0.40	0.19	0.17	−0.47	0.27
MAP	0.35	0.19	−0.47	0.31	0.10	0.15	−0.48	0.25	−0.39	−0.41	0.26	0.06
MSP	0.37	0.19	−0.50	0.32	0.09	0.18	−0.51	0.26	−0.43	−0.48	0.27	0.00
AHM	−0.35	−0.23	0.54	−0.27	−0.12	−0.24	0.63	−0.32	0.34	0.38	−0.28	0.02
SHM	−0.36	−0.30	0.48	−0.30	−0.20	−0.33	0.58	−0.32	0.39	0.37	−0.29	0.04
FFP	0.20	−0.32	0.18	0.13	−0.05	−0.43	0.24	−0.11	0.04	−0.22	−0.20	−0.06
PAS	0.01	0.20	−0.53	−0.01	−0.07	0.21	−0.50	0.08	−0.31	−0.21	0.13	0.20
ELEV	−0.26	0.29	−0.33	−0.29	0.04	0.43	−0.39	0.04	0.00	0.17	0.23	0.07
LAT	0.36	−0.34	0.00	0.38	−0.17	−0.33	0.08	0.15	−0.01	−0.38	−0.20	−0.24
LON	−0.06	0.03	−0.14	−0.08	−0.01	−0.10	−0.18	−0.11	−0.18	−0.13	−0.22	0.20

* Correlations >±0.18 are significantly different from zero at *P* = 0.05.

† See Table 1 for trait codes and descriptions.

‡ Climate and geography variables are MAT, mean annual temperature; MWMT, mean warmest month temperature; MCMT, mean coldest month temperature; TD, temperature difference between MWMT and MCMT or continentality; MAP, mean annual precipitation; MSP, mean summer precipitation; AHM, annual heat/moisture index (MAT+10)/(MAP/1000); SHM, summer heat/moisture index (MWMT+10)/(MSP/1000); FFP, frost-free period; PAS, precipitation as snow; ELEV, elevation; LAT, latitude; LON, longitude.

**Figure 2:**
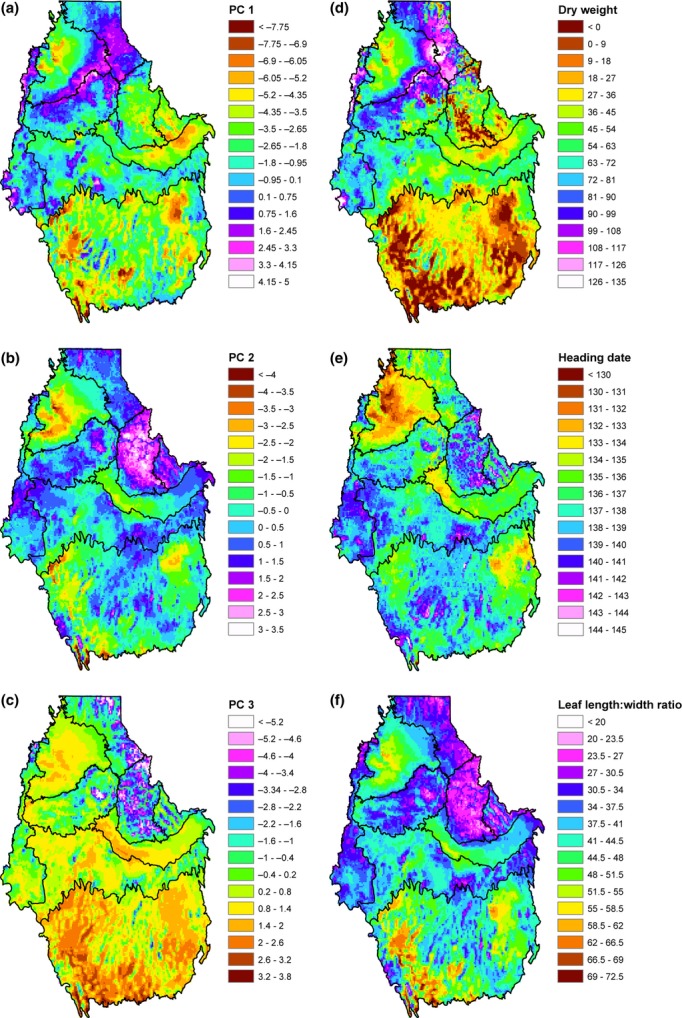
Geographic variation in the first three principle components (A–C) and related individual traits of dry weight (D), heading date (E), and leaf height/width ratio (F).

Several traits as measured individually or as multivariate traits were related to climate as indicated by moderate correlations (Table 6) and coefficients of determination (*R*^2^) from regressions (Table 7). Maps of geographic variation produced from regression equations corresponded closely with large physiographic features across the landscape including level III ecoregions (Fig. 2). Nevertheless, gradients in traits existed within ecoregions corresponding to gradients in temperature, precipitation, and aridity. As might be expected from the correlations between PCs and individual traits, landscape patterns seen in PC 1 were very close to patterns seen in dry weight; patterns seen in PC 2 were close to patterns seen in heading date; and patterns seen in PC 3 were close to patterns seen in leaf form. In general, populations from areas with mild climates, including relatively warm winters, low seasonal temperature differentials, high precipitation, and low aridity, were larger and more vigorous as measured by dry weights and higher values of PC 1 and were generally greener and had longer awns. Maps of PC 1 and dry weight indicate that these areas were the relatively mild areas of the Blue Mountains, northern Rockies in Idaho, and the eastside Cascades in Oregon. Plants from arid areas such as the Columbia Plateau, the Snake River Plain, and central and eastern Idaho were smaller and less vigorous. That was also the case in much of the lower elevations of the central Basin and Range, but many of these areas appear to be outside of the realized niche of the species (based on observations during collections). Plants from cold and dry areas of the Great Basin and central Idaho tended to be intermediate in size. Populations with later phenology as measured by later heading dates and higher values of PC 2 were from areas that were colder, particularly in the winter, with a shorter frost-free period and somewhat more arid. These areas tended to be in central and eastern Idaho, the eastern Great Basin, and the eastside Cascades. Earlier phenology was found in populations from the warm and arid Columbia Plateau. Populations with narrow leaves, whether measured as leaf width or the ratio of leaf length to width, and correspondingly high values of PC 3 were from hot, arid climates with low precipitation, particularly in the summer. The narrowest leaves came from areas of the Columbia Plateau and the lower Snake River Plain, and the widest leaves came from mountainous areas, especially in Idaho and the Blue Mountains. Populations from arid climates with low precipitation also produced plants with leaves that were more pubescent and had a more upright growth habit.

**Table 7:** Regression equations and coefficients of determination (*R*^2^) for regression of principle components scores and traits on seed source climate

Trait*	*R*^2^	Regression equation†
PC1	0.41	17.12 + 0.02 × TD − 0.02 × SHM + 0.47 × EMT
PC2	0.28	3.37 + 0.02 × TD − 0.007 × SHM − 0.02 × FFP
PC3	0.35	−2.07 − 0.004 × PAS + 0.004 × CMD
DRYWT	0.51	1138.87 − 13.51 × MWMT + 7.24 × TD + 0.05 × MAP − 0.16 × SHM −3.2 × bFFP − 1.91 × FFP − 0.2 × PAS + 7.9 × EMT
HEAD	0.33	147.07 − 5.34 × MAT + 2.6 × MCMT + 1.48 × TD + 0.53 × EMT + 0.03 × Eref
LFRATIO*	0.44	20.33 + 1.11 × MAT + 0.2 × AHM

* See Table 1 for trait codes and descriptions; PC, principle component.

† Climate variables are MAT, mean annual temperature; MWMT, mean warmest month temperature; MCMT, mean coldest month temperature; TD, temperature difference between MWMT and MCMT or continentality; MAP, mean annual precipitation; AHM, annual heat/moisture index (MAT+10)/(MAP/1000); SHM, summer heat/moisture index (MWMT+10)/(MSP/1000); FFP, frost-free period; bFFP, beginning of frost-free period; PAS, precipitation as snow; EMT, extreme minimum temperature over 30 years; Eref, Hargreaves reference evaporation; CMD, Hargreaves climatic moisture deficit.

### Seed zone delineation

**Figure 3:**
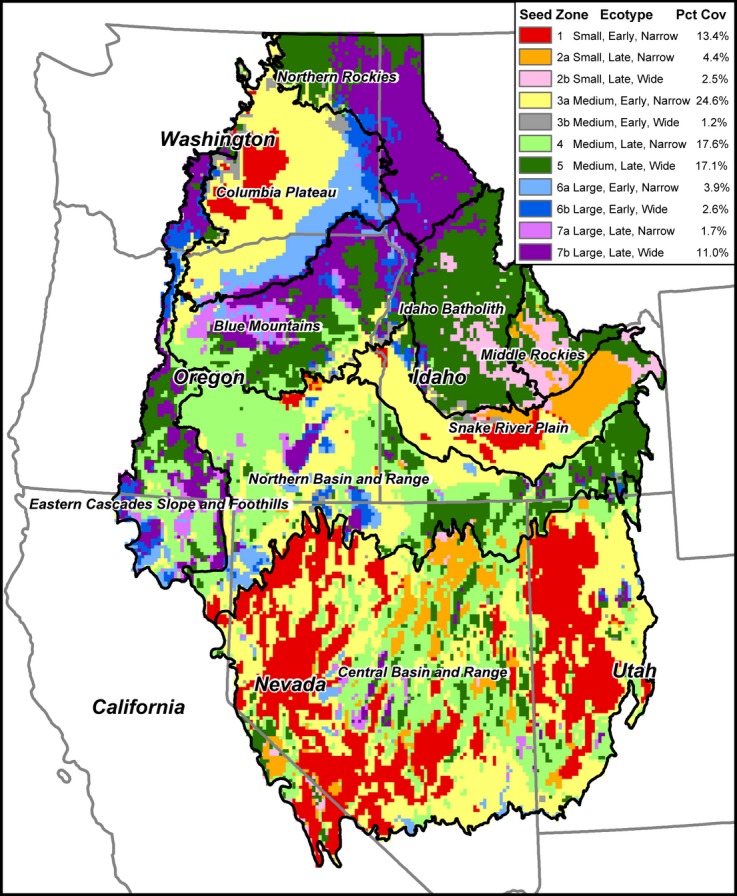
Recommended seed zones (labeled 1–7b) for *Pseudoroegneria spicata* in the inland Pacific Northwest. Ecotypes correspond to small, medium, or large plant size; early or late phenology; and narrow or wide leaf width. Percentages of total area within study area for each seed zone are given in the legend.

Seed zones were delineated by classifying the scores of the first PC into three categories and classifying the scores of the second and third PCs into two categories, and then overlapping the maps of the first three PCs (Fig. 3). Three categories were chosen for the first PC because the higher *R*^2^ of the regression on climate and the larger amount of variation explained in the PCA indicate that it is likely of greater importance for adaptation than the next two PCs. Although twelve seed zones are possible by overlapping these three maps, one of the seed zones is not present, the seed zone representing low values of PC 1, PC 2, and PC 3. In other words, ecotypes with the combination of traits of small size, early phenology, and wide leaves did not occur in the area of the study. Four other seed zones were rare with less than 3 percent of the study area occupied by each zone. These included areas of populations with large plants, late phenology, and narrow leaves (zone 7a); populations with large plants, early phenology, and wide leaves (zone 6b); populations with intermediate-sized plants, early phenology, and wide leaves (zone 3b); and populations with small plants, late phenology, and wide leaves (zone 2b). Thus, most of the study area is represented by seven seed zones. Indeed, three of the seed zones with ecotypes of intermediate-sized plants represent the majority of the study area (59 percent) including much of the Columbia Plateau, lower elevations of the Great Basin, central Idaho, and the eastside Cascades,. The seed zone classification scheme chosen included eleven seed zones with the seven most common seed zones labeled 1–7. The four rare seed zones were included as a classification within one of the seven zones in which the first two PCs are similar, but the third PC differs in the relative widths of leaves. Zones labeled ‘a’ represent zones with ecotypes with narrow leaves, whereas zones labeled ‘b’ represent zones with ecotypes with wide leaves. Rare zones generally represented unusual combinations of traits.

### Comparisons of commercial releases with native sources

Releases were more vigorous compared with the average of the native sources when grown together in the common environments of the test sites in this study (Table 8), but similar in size to the native sources near their origin of the eastern edge of the Columbia Plateau (Figs 1 and 2). Releases were considerably larger and more fecund, had greater regrowth after harvest, and had larger leaves and inflorescences. Although all releases were more vigorous, considerable variation was found among them. Whitmar was smaller and Secar was larger than the other releases. Phenology was similar between the releases and the native sources, although variation was found among them. Secar had later dates of heading and anthesis, but seed matured sooner than the other releases and the native sources. Anatone had a somewhat earlier phenology. Considerable variation was found in the length of awns. As expected, Whitmar, also known as ‘beardless wheatgrass’ because of its awnless form, had scores for awn length near one. Anatone and P-7 also had low scores for awn length. Secar was characterized by relatively long awns. Secar was also characterized by low leaf pubescence and a more upright plant form. Other releases were less upright than the native sources. Leaf ratio was similar between releases and native sources.

**Table 8:** Overall means for native collections, five commercial releases, and the mean of five commercial releases

Trait*	Native sources	All releases	Whitmar	Goldar	Anatone	P-7	Secar
DRYWT	75	160	132	156	150	159	201
CW	8.2	11.5	10.9	11.5	11.2	11.6	12.5
HT	36	43	40	39	41	44	51
REGRWT	24	47	35	48	42	50	58
REGRHT	14	19	17	17	18	19	24
INFLNO	87	177	166	167	194	157	201
CULMLNG	43	50	48	45	48	52	57
SPKLNG	16	18	19	17	18	19	15
INFLLNG	59	68	67	62	65	71	73
SPKNO	10.5	13.1	13.5	12.1	11.7	13.6	14.8
LFLNG	14.0	15.9	15.4	16.1	16.2	16.4	15.3
LFWD	0.46	0.52	0.45	0.58	0.55	0.49	0.52
LFRATIO	38	37	42	32	39	41	34
LFCOL	3.1	3.5	3.7	3.5	3.3	3.6	3.4
LFPUB	4.1	3.9	4.2	4.2	4.3	4.0	2.9
HABIT	6.3	5.8	5.4	5.5	5.7	5.7	6.5
AWNS	4.6	2.9	1.1	3.8	1.9	1.7	6.2
HEAD	137	139	138	140	136	139	142
BLOOM	153	154	154	151	152	154	158
MATURE	191	191	192	192	191	193	189
GERM	6.39	5.68	6.17	6.19	5.82	5.02	5.15
SEEDWT	0.40	0.37	0.32	0.41	0.45	0.40	0.28

* See Table 1 for trait codes and descriptions.

## Discussion

Large and significant variation among populations associated with climates at seed sources was found for *P. spicata* plants grown in a common environment. Furthermore, the relationship of traits with climates makes sense for adaptation. As expected, phenotypic plasticity was found for traits related to size, fecundity, and phenology as indicated by significant differences among test sites and years; plants grown in warmer, drier test environments were larger and more fecund and had an earlier phenology, and phenology was earlier in 2007 than in 2008. Nevertheless, significant variation among populations associated with climates at seed sources is indicative of genetic variation that is not plastic, but rather the result of natural selection to conditions at seed source locations (Reich et al. 2003). In other words, if populations were perfectly phenotypically plastic in response to environments, then natural selection would not be necessary for adaptation, and we would not find differences among populations grown in a common environment.

Correlations and coefficients of determination (*R*^2^) values from regressions indicated moderate levels of adaptation of *P. spicata* populations to multivariate climates when compared to other species. *R*^2^ values for traits regressed on climate were between 0.51 and 0.28 (Table 7), compared with Douglas-fir (*Pseudotsuga menziesii*), for example, which had *R*^2^ values of 0.68 and 0.50 for the first two multivariate traits in a canonical correlation analysis (St. Clair et al. 2005), and is considered to be closely adapted to environments when compared with other conifer species (Rehfeldt 1994b). The R^2^ values found in the current study are similar in magnitude to those found for two genecology studies of grass species over a more restricted area in the Blue Mountains: *E. glaucus* with *R*^2^ values between 0.51 and 0.30 for the first four PCs (Erickson et al. 2004) and *B. carinatus* with *R*^2^ values between 0.46 and 0.40 for the first two PCs (Johnson et al. 2010a,b).

The relationships between traits and climates found in this study are consistent with those of other studies of grass species with a summer growing season and make sense from an adaptation perspective. *Pseudoroegneria spicata* populations from warm, arid source environments were smaller with earlier phenology, relatively narrow leaves and greater leaf pubescence (Table 6). Erickson et al. (2004) in the study of *E. glaucus* of the Blue Mountains ecoregion found shorter plants; earlier flowering; and narrow, more pubescent leaves, as well as more rapid germination, in populations from more arid climates. Studies of other grasses in the same region, however, found contradictory results. Johnson et al. (2010a,b) found later phenology and larger *B. carinatus* plants from populations from warmer, drier climates, and Parsons et al. (2011) found later phenology from *Elymus elymoides* plants from warmer climates. Johnson et al. (2010a,b) suggested that later flowering in warmer climates allowed more time for leaf and tiller growth before partitioning plant resources to reproduction, whereas in cooler climates, flowering was earlier to allow for a shortened growing season. *Bromus carinatus* does not occur in habitats as dry as those of *P. spicata*; thus, competition for water may not be as important as competition for light for *B. carinatus* populations at the drier, warmer end of the species niche. Our findings for within-species variation in *P. spicata* are consistent with findings from studies looking at correlations of variation among species and climate. Species from hot, dry, nutrient-poor environments tend to be smaller, shorter, and with thicker, narrower, more pubescent leaves, whereas species from wetter, higher nutrient environments tend to be bigger, taller, and have larger leaves (Hendrix et al. 1991; Holmes and Keiller 2002; Westoby et al. 2002; Moles et al. 2009). Water loss from evapotranspiration in arid climates may be expected to be less from plants with less shoot biomass, smaller stature, greater leaf pubescence, and narrower leaves with greater leaf mass per area. Competitive ability in arid systems may be enhanced by smaller size and greater allocation of biomass to shoots versus roots (Rowe and Leger 2011). Earlier reproductive phenology may also be advantageous in the arid, summer-drought climates found in the intermountain western United States because plants are able to complete critical reproductive processes before the onset of drought. In contrast, photosynthetic capacity and competitive ability in mild, less stressful climates may be expected to be greater for plants of greater biomass, taller stature, and wider leaves. In much colder and drier climates, however, conservative growth may again become an important adaptive strategy. Thus, growth potential might be expected to be less for populations from cold, dry habitats of the eastern Great Basin and central Idaho than for populations from the wetter, milder climates in northern Idaho and the Blue Mountains.

The five commercial releases were considerably larger and more fecund than most of the native ecotypes (Table 8). Four of the releases were derived from selection among seed collected in southwestern Washington (Monsen et al. 2004; Ogle et al. 2010; Fig. 1). Anatone is harvested from a population originating from Asotin County, Washington, at an elevation of 975–1100 m. Goldar was selected from a population collected in the Umatilla National Forest, also in Asotin County, Washington, at an elevation of 310–475 m. Whitmar was selected from a population native to the Palouse grasslands near Colton, Washington. Secar was originally thought to be of the species *P. spicata*, but was later determined to be of the species *Elymus wawawaiensis* (Snake River wheatgrass) (Carlson and Barkworth 1997). It is derived from an early collection made in 1938 near Lewiston, Idaho. Although a comparison with native ecotypes of *P. spicata* may not be appropriate, Secar appears to be well adapted to low-elevation sites in the intermountain western United States (Monsen et al. 2004), including the test sites in this study. P-7 is a multiple-origin polycross generated by intermating plants from 23 open-pollinated native collections and two releases (Goldar and Whitmar) from Washington, Oregon, Nevada, Utah, Idaho, Montana, and British Columbia (Jones et al. 2002). None of the germplasm present in P-7 is from the more arid regions of the Columbia Plateau and Snake River Plain. Given the origins of all five releases in areas of high growth potential for the species, it is not surprising that they grew well in this study, similar to the nearby collections in the case of single-origin releases (Fig. 2). Although some selection pressure for high growth and fecundity may have been applied during the process of growing and harvesting the selections, much of the increase in size and fecundity may be attributed to among-population selection. Although the increased vigor of the releases might be desirable from a standpoint of revegetating a site to avoid soil erosion or to provide forage, it is not yet clear that these releases are the best choice for restoration of grassland ecosystems over a wide area. Paradoxically, large size may be a disadvantage when competing against invasive species. Rowe and Leger (2011) found that *Elymus multisetus* populations in areas invaded by *B. tectorum* (cheatgrass) were evolving to be more competitive against *B. tectorum* and that the characteristics of *E. multisetus* plants that confer competitiveness included smaller size, greater allocation of biomass to roots, and higher percentage of fine roots. Competition appears to be primarily for belowground resources rather than light. Thus, not only might small size be advantageous for adaptation of *P. spicata* to arid climates, it might also be advantageous for competition against *B. tectorum*. Another concern about the use of releases is the potential for genetic swamping of local populations during large-scale restoration (Montalvo et al. 1997; Hufford and Mazer 2003; McKay et al. 2005), particularly given the higher fecundity of releases. The potential for evolution in response to new stresses, including invasives and climate change, depends on within-population genetic variation, which could be reduced by the widespread use of releases with limited variation compared with populations mixtures from within seed zones.

We propose a seed zone delineation that includes eleven seed zones with some zones further divided into areas of ecotypes with narrow or wide leaves and designated as “a” and “b” (Fig. 3). The number of seed zones seems to be a reasonable compromise between ensuring adapted plant material and allowing for bulking of populations for use over fairly large areas to provide some economies of scale in seed production and storage. The suggested number of seed zones is similar to that suggested for the Blue Mountains for two other grass species; we propose six seed zones for most of the Blue Mountains, whereas four seed zones were proposed for *E. glaucus* (Erickson et al. 2004) and for *B. carinatus* (Johnson et al. 2010a,b), but over an area that excluded lower elevations. Our suggested seed zone delineation may be too conservative for some seed producers or land managers who are looking for greater economies of scale, while it might be too risky for others who are concerned about maladaptation and conservation of native genetic diversity. The National Park Service, for example, has a policy of using seed from ‘populations as closely related genetically and ecologically as possible to park populations, preferably from similar habitats in adjacent or local areas’ (National Park Service 2006). Land managers that wish to be more conservative with respect to maintaining adaptation and local genetic structure may choose to use eleven instead of seven seed zones and may further divide the region into seed zones that include level III ecoregions; that is, they might avoid moving populations between level III ecoregions even if they occur in the same seed zones as indicated in Fig. 3. Another option might be to bulk seed from a limited area in a seed zone within a level III ecoregion and make the seed from the local ecoregion the first priority for restoration. When seed supplies become limited, however, managers might consider using collections from the same seed zone in adjacent level III ecoregions.

## Conclusions

Results from this study indicate that *P. spicata* populations differ in traits important for adaptation. Several traits, particularly those associated with size, flowering phenology, and leaf width, showed considerable population variation when populations were grown together in a common environment. Furthermore, that variation was related to the climates of seed sources in ways that made sense for survival, growth and reproduction, and thus adaptation. Seed zones that take into account the different ecotypes across the landscape may help ensure that material used in restoration is adapted to climates at the restoration sites. We recommend, however, the establishment of reciprocal transplant studies to better evaluate local adaptation of ecotypes as indicated by our proposed seed zone delineation. Populations should be selected from each of the seven to eleven seed zones and grown at test sites within each of seed zones. Of particular interest would be the establishment, growth, survival, and reproduction of populations from mesic habitats with moderate temperatures, such as those from seed zones 6 or 7, when planted in warm, arid climates, such as in seed zone 1, and the reciprocal transplant. Transfers to much colder and drier climates would also be of interest. Although adaptation as measured by persistence over generations is of greatest interest, such a study should also measure components of fitness such as phenology and leaf width or leaf mass area. Establishment might be measured by sowing seed at each site and following emergence capacity and rate. An evaluation of releases grown in each of the seed zones in comparison with local ecotypes would also be of interest and should help evaluate the climatic niche of each release. Finally, it might also be valuable to look at competitive interactions of releases and local ecotypes with *B. tectorum* using a study design such as that of Rowe and Leger (2011).

## Data archiving statement

Data deposited in the Dryad repository: doi:10.5061/dryad.sr4rm.

## Supporting Information

**Table S1.** Correlations of population means between test sites in two different years and between years at each test site for traits measured on 114 *Pseudoroegneria spicata* populations.

**Table S2.** Correlations of first three principle components with individual traits averaged over sites and years.
