# Supplementary material for: Genetic variation in adaptive traits and seed transfer zones for Pseudoroegneria spicata (bluebunch wheatgrass) in the northwestern United States
Source: Evol Appl. 2013 Jun 6;6(6):933–48. doi: 10.1111/eva.12077 (PMC3779094; doi:10.1111/eva.12077)
Supplement: Supplementary file 1 [file eva0006-0933-SD1.docx]

Table S1. Correlations of population means between test sites in two different years and between years at each test site for traits measured on 114 *Pseudoroegneria spicata* populations.

|  | Correlations^2^ between test sites^3^ | | | | | | |  |  |  |
| --- | --- | --- | --- | --- | --- | --- | --- | --- | --- | --- |
|  | in 2007 | | | | in 2008 | | | Correlations^2^ between years at | | |
| Trait^1^ | CF-LP | | CF-PU | LP-PU | CF-LP | CF-PU | LP-PU | CF | PU | LP |
| DRYWT | 0.84 | 0.79 | | 0.80 | 0.81 | 0.81 | 0.76 | 0.77 | 0.90 | 0.93 |
| CW | 0.83 | 0.79 | | 0.75 | 0.80 | 0.81 | 0.77 | 0.84 | 0.90 | 0.88 |
| HT | 0.45 | 0.52 | | 0.51 | 0.55 | 0.54 | 0.58 | 0.34 | 0.79 | 0.60 |
| REGRWT | 0.67 | 0.73 | | 0.72 | 0.64 | 0.71 | 0.72 | 0.68 | 0.85 | 0.87 |
| REGRHT | 0.50 | 0.56 | | 0.59 | 0.57 | 0.63 | 0.65 | 0.65 | 0.75 | 0.72 |
| INFLNO | 0.85 | 0.82 | | 0.78 | 0.65 | 0.76 | 0.55 | 0.78 | 0.71 | 0.77 |
| SPKNO | 0.75 | 0.75 | | 0.78 | 0.70 | 0.73 | 0.70 | 0.62 | 0.74 | 0.71 |
| CULMLNG | 0.29 | 0.68 | | 0.42 | 0.41 | 0.71 | 0.55 | 0.67 | 0.68 | 0.84 |
| SPKLNG | 0.18 | 0.20 | | 0.69 | 0.57 | 0.58 | 0.57 | 0.18 | 0.54 | 0.64 |
| INFLLNG | 0.26 | 0.33 | | 0.55 | 0.45 | 0.70 | 0.61 | 0.27 | 0.64 | 0.82 |
| LFLNG | 0.70 | 0.65 | | 0.73 | 0.46 | 0.65 | 0.55 | 0.60 | 0.65 | 0.63 |
| LFWD | 0.85 | 0.54 | | 0.48 | 0.69 | 0.67 | 0.63 | 0.69 | 0.88 | 0.37 |
| LFRATIO | 0.83 | 0.84 | | 0.83 | 0.79 | 0.81 | 0.76 | 0.78 | 0.86 | 0.80 |
| LFCOL | 0.84 | 0.45 | | 0.40 | 0.65 | 0.51 | 0.51 | 0.69 | 0.91 | 0.27 |
| LFPUB | 0.51 | 0.85 | | 0.62 | 0.42 | 0.72 | 0.26 | 0.84 | 0.67 | 0.75 |
| HABIT | 0.61 | 0.52 | | 0.42 | 0.74 | 0.65 | 0.65 | 0.62 | 0.72 | 0.39 |
| AWNS | 0.87 | 0.89 | | 0.90 | 0.86 | 0.82 | 0.90 | 0.83 | 0.95 | 0.88 |
| HEAD | 0.86 | 0.72 | | 0.70 | 0.69 | 0.52 | 0.49 | 0.71 | 0.82 | 0.42 |
| BLOOM | 0.79 | 0.69 | | 0.75 | 0.38 | 0.22 | 0.64 | 0.30 | 0.83 | 0.60 |
| MATURE | 0.53 | 0.39 | | 0.50 | 0.19 | 0.01 | 0.51 | 0.05 | 0.52 | 0.21 |
| GERM^4^ | - | - | | - | 0.38 | 0.30 | 0.27 | - | - | - |
| SEEDWT^4^ | - | - | | - | 0.52 | 0.42 | 0.55 | - | - | - |

^1^See Table 1 for trait codes and descriptions.

^2^ Correlations > ±0.18 are significantly different from zero at p=0.05.

^3^CF = Central Ferry, LP = Lucky Peak, PU = Pullman

^4^ Germination and seed weight traits were measured in 2008 only

Table S2. Correlations^1^ of first three principle components with individual traits averaged over sites and years.

| Trait^2^ | PC1 | PC2 | PC3 |
| --- | --- | --- | --- |
| DRYWT | 0.94 | 0.02 | 0.06 |
| CW | 0.93 | -0.16 | -0.07 |
| HT | 0.56 | 0.57 | 0.34 |
| REGRWT | 0.91 | -0.05 | 0.07 |
| REGRHT | 0.68 | -0.08 | 0.55 |
| INFLNO | 0.83 | -0.13 | 0.16 |
| CULMLNG | 0.72 | 0.32 | 0.20 |
| SPKLNG | 0.21 | 0.11 | 0.03 |
| INFLLNG | 0.63 | 0.29 | 0.16 |
| SPKNO | 0.63 | 0.28 | 0.23 |
| LFLNG | 0.48 | 0.41 | 0.43 |
| LFWD | 0.47 | 0.15 | -0.53 |
| LFRATIO | -0.24 | -0.06 | 0.73 |
| LFCOL | 0.55 | 0.11 | -0.28 |
| LFPUB | -0.40 | -0.31 | 0.26 |
| HABIT | -0.58 | -0.07 | 0.44 |
| AWNS | 0.21 | 0.13 | -0.32 |
| HEAD | -0.07 | 0.82 | -0.13 |
| BLOOM | -0.11 | 0.87 | 0.18 |
| MATURE | 0.06 | 0.58 | -0.13 |
| GERM | -0.40 | 0.51 | 0.06 |
| SEEDWT | -0.19 | -0.01 | -0.04 |

^1^Correlations > ±0.18 are significantly different from zero at p=0.05.

^2^See Table 1 for trait codes and descriptions.
